# Impact of Ageism on Civic Engagement and Mental Health Among Older Adults: A Qualitative Study

**DOI:** 10.1192/j.eurpsy.2024.713

**Published:** 2024-08-27

**Authors:** S. von Humboldt, A. Costa, N. Ilyas, I. Leal

**Affiliations:** ^1^William James Center for Research, ISPA – Instituto Universitário; ^2^ISPA – Instituto Universitário, Lisbon, Portugal; ^3^Center for Clinical Psychology, University of the Punjab, Lahore, Pakistan

## Abstract

**Introduction:**

Ageist beliefs and attitudes may restrict the opportunities for older adults to participate actively in their communities, resulting in strong effects on mental health.

**Objectives:**

This study has three objectives: 1) To investigate the effect of ageism on older adults’ civic activities; 2) To analyze the influence of ageism on mental health; and 3) To explore the impact of civic participation on older adults’ mental health.

**Methods:**

This qualitative study included 391 older people from three different nationalities (Portuguese, Brazilian and English) ranging in age from 65 to 88 years old. All the interviews went through the process of content analysis.

**Results:**

For the first objective, findings encompass four major themes: (1) Social disapproval (86%); (2) Perceived Ineptitude (84%); (3) Anticipated Failure (83%); and (4) Inability to Contribute (77%). For the second objective, findings indicated six categories: (1) Helplessness and Despair (89%); (2) Rage (81%); (3) Self-Perceived Inability (77%); (4) Sense of Unimportance (71%); (5) Anxiety (68%); and (6) Outbursts of Emotion (63%). For the third objective, the following five major subjects emerged: (1) Meaningfulness (81%); (2) Embracing Social Belonging (80%); (3) Cognitive Abilities (71%); (4) Personal Empowerment (67%); (5) Emotional Expression (54%). Additionally, findings indicated that the most verbalized themes for the three objectives were the same across the three nationalities.

**Conclusions:**

The results of this study offered insight into how ageism, mental health, and civic engagement are related. Ageism seems to have a negative impact on mental health. Ageism also made it difficult for people to participate in civic life, which has been linked to better mental health. These findings emphasize the need to identify ageism and encourage inclusive civic involvement to improve older individuals’ mental health.

Keywords: Mental health; ageism; civic participation; older adults.

**Disclosure of Interest:**

None Declared

